# Combining Clinical, Pathological, and Demographic Factors Refines Prognosis of Lung Cancer: A Population-Based Study

**DOI:** 10.1371/journal.pone.0017493

**Published:** 2011-02-25

**Authors:** Joseph Putila, Scot C. Remick, Nancy Lan Guo

**Affiliations:** 1 Mary Babb Randolph Cancer Center, West Virginia University, Morgantown, West Virginia, United States of America; 2 Department of Community Medicine, West Virginia University, Morgantown, West Virginia, United States of America; 3 Department of Medicine, West Virginia University, Morgantown, West Virginia, United States of America; University Medical Center Rotterdam, Netherlands

## Abstract

**Background:**

In the treatment of lung cancer, an accurate estimation of patient clinical outcome is essential for choosing an appropriate course of therapy. It is important to develop a prognostic stratification model which combines clinical, pathological and demographic factors for individualized clinical decision making.

**Methodology/Principal Findings:**

A total of 234,412 patients diagnosed with adenocarcinomas or squamous cell carcinomas of the lung or bronchus between 1988 and 2006 were retrieved from the SEER database to construct a prognostic model. A model was developed by estimating a Cox proportional hazards model on 500 bootstrapped samples. Two models, one using stage alone and another comprehensive model using additional covariates, were constructed. The comprehensive model consistently outperformed the model using stage alone in prognostic stratification and on Harrell's C, Nagelkerke's R^2^, and Brier Scores in the whole patient population as well as in specific treatment modalities. Specifically, the comprehensive model generated different prognostic groups with distinct post-operative survival (log-rank *P*<0.001) within surgical stage IA and IB patients in Kaplan-Meier analyses. Two additional patient cohorts (*n* = 1,991) were used as an external validation, with the comprehensive model again outperforming the model using stage alone with regards to prognostic stratification and the three evaluated metrics.

**Conclusion/Significance:**

These results demonstrate the feasibility of constructing a precise prognostic model combining multiple clinical, pathologic, and demographic factors. The comprehensive model significantly improves individualized prognosis upon AJCC tumor staging and is robust across a range of treatment modalities, the spectrum of patient risk, and in novel patient cohorts.

## Introduction

Lung cancer is one of the most aggressive cancer types and consistently the leading cause of cancer-related death in the United States for both men and women. There are around 215,000 new cases and 161,000 deaths annually [1]. Non-small cell lung cancer (NSCLC) accounts for about 80% of lung cancer cases. Although tumor stage is strongly predictive of survival in most cases, it does not explain the distinct variability in treatment outcome within patients of the same stage. Currently, surgery is the major treatment option for patients with stage I NSCLC. However, 35–50% of stage I NSCLC patients will relapse within five years [2], [3], which is the major cause of treatment failure, i.e. death from lung cancer. It remains an unsolved challenge for physicians to reliably identify patients at high risk for tumor recurrence as candidates for adjuvant chemotherapy.

Recent studies have utilized a variety of information in addition to tumor stage for prognostic stratification and prediction of treatment outcome [4]–[12]. Prognostic factors such as age, gender, and tumor grade, have been shown to be strongly associated with survival. Age is a well established risk factor for the development of lung cancer and can also influence the type of treatment received either due to medical coverage or the existence of co-morbid conditions which preclude certain therapies [13], [14]. Males diagnosed with lung cancer consistently experience poorer survival than do females [15]. This gender difference persisted even when controlling for other variables such as tumor stage, age at diagnosis, and treatment.

Race has also been shown to be a significant predictor of survival, with Asians and Pacific Islanders experiencing better survival in both prospective [16] and population-based studies [17]. While the disease mechanism and genetic background is not well characterized, the consistency of this finding is useful in terms of prognostication and treatment.

The emerging use of genetic markers may enable physicians to make treatment decisions based on the specific characteristics of individual patients and their tumors, instead of population statistics [18]. This study presents an alternative avenue to improve personalized prognosis of NSCLC by combining clinical, pathological, and demographic factors in a population-based study (*n* = 234,412). This comprehensive model was tested across a number of treatment modalities and blindly validated on multiple separate patient cohorts (*n* = 1,991). The comprehensive model achieved a significant improvement in prognostication when compared with AJCC tumor staging system including cases converted to AJCC 7^th^ Edition [19]. This patient stratification scheme could be integrated with future clinically-validated prognostic gene signatures for personalized prognosis of NSCLC.

## Methods

### Acquisition of Patient Cohorts

A cohort of patients diagnosed with lung cancer was retrieved from the Surveillance Epidemiology and End Results (SEER) database [20]. The SEER database is an aggregate of registry data from specific geographic areas covering approximately 26 percent of the U.S. population, and contains clinical, demographic, treatment, and follow-up information for a variety of cancers. The requirements for inclusion in the study included a diagnosis of primary lung adenocarcinoma (ICD-O-3 8140 to 8380) or squamous cell carcinoma (ICD-O-3 8050 to 8080) between the years 1988 and 2006, as well as available data on tumor stage, tumor grade, race, age, gender, disease-specific survival, and treatment. Patients who were diagnosed via autopsy or death certificate, or had no valid survival data were excluded from the analysis. A total of 234,412 patients met the inclusion criteria. Patients staged using the 6^th^ edition of AJCC staging, in general 2004 and newer diagnoses, were recoded to the 7^th^ edition based on the proposed staging changes in the AJCC Staging Manual [19] and information about tumor size, extension, metastasis, and lymph node involvement found in the SEER database where possible. A total of 58,634 cases were able to be converted from the 6^th^ to the 7^th^ edition.

Two additional patient cohorts were also used as validation sets. De-identified data for a total of 1,552 patients treated at the Mary Babb Randolph Cancer Center at West Virginia University from 1990 to 2009 with squamous cell carcinoma (*n* = 758) or adenocarcinoma (*n* = 794) were obtained. The study was approved with an IRB exemption from West Virginia University. According to HIPAA regulation, de-identified clinical information can be used in research without prior consent from the patients. A total of 439 lung adenocarcinoma cases were also obtained from Shedden et al [21] for patients with Stage I-IIIB cancers. These patients were treated in H. Lee Moffitt Cancer Center, University of Michigan Comprehensive Cancer Center, Dana-Farber Cancer Institute, and Memorial Sloan-Kettering Cancer Center. Patients have provided consent. These data have been published in Shedden et al [21] before. It is not clear if patients have provided written or verbal consent. The protocols were approved with Institutional Review Boards (IRB-Med) of the respective institutes.

### Conversion of Cases to AJCC 7^th^ Edition

Cases diagnosed from 2004 onward were able to be converted into the AJCC 7^th^ Edition. The original TNM staging information regarding tumor size and extension (T), lymph node status (N), and distant metastasis (M) was retrieved from the SEER data. Using this information, the T, N, and M classifiers were recoded according to the new guidelines [19] and then used to determine the AJCC 7^th^ Edition stage.

### Model Construction and Statistical Analyses

Disease-specific survival was analyzed primarily using a Cox proportional hazards model. This model estimates the effect of covariates on the time until an event, in this case death, following a diagnosis. Four models, one for each of the histology and AJCC staging combinations, were estimated. A total of 500 bootstrapped samples equal in size to the original adenocarcinoma and squamous cell carcinoma patient cohorts were constructed. This method has been seen to be superior to split-sample techniques [22], and in general produces less biased estimates with a smaller variance. A Cox model was then fit on each bootstrapped sample. In order to determine the advantage of using other covariates in addition to AJCC stage, two sets of covariates were used in the model evaluation. The first contained information on tumor stage and grade, patient age, race, and gender. The second contained only information on tumor stage and was used as a model of current clinical practice. The final training model used the mean value of all coefficients generated from the bootstrapped samples, as the distribution of hazard scores was normal. Hazard scores were calculated for each patient in the original samples based on the final model constructed from the means. The formula used to specify the model is shown below, demonstrating the relationship between hazard *h* for patient *i* at time *t* and the coefficients, β, for covariates 1 through *k* with values of *x*.
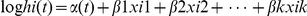



In the prognostic categorization, cutoff values were defined from the bootstrapped samples to stratify patients into a high-, low-, or intermediate-risk group based on their individual hazard scores. The Cox-model and cutoff values were applied to the original cohort for validation. The prognostic categorization was evaluated with the Kaplan-Meier survival function, where the estimated proportion surviving *S* at any time *t* is equal to the proportion of non-censored cases *n* surviving interval *i* less the number of deaths *d* in that interval, as in the following formula:
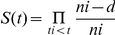



Patients still alive or dead due to unrelated causes were censored at the time of last follow-up or death, respectively. Internal performance was measured using Harrell's C, Nagelkerke's R^2^, and Brier Scores. Harrell's C is a measure of concordance which is representative of the area under an ROC curve ranging between 0 and 1, with higher scores indicating greater concordance [23]. The ROC curves were used in model evaluation with the *pROC* package in R. The statistical significance (*P-*value) of the difference between the areas under the curves was calculated using the Delong method in the same package. A larger area in this case demonstrates an improved predictive ability. Nagelkerke's R^2^ is functionally similar to the R^2^ value in linear models, ranging between 0 and 1 with higher values explaining more variance, with this variant being calculated on the log-likelihood scale. The Brier score represents the average prediction error, ranging from 1 to 0, with lower values indicating a lower average error. Significance of risk-group stratification was determined using a log-rank test of the Kaplan-Meier function. The log-rank test uses contingency tables at each observation period to determine if a significant difference exists between two survival functions. The model constructed using the training set was then further validated on SEER sub-cohorts as well as patients from the MBRCC and the Director's Challenge cohorts [21], without re-estimating parameters of the model or cutoffs. Statistical analyses were conducted with the *pamr*, *pec*, *Design*, and *survival* packages in *R* v2.11.0.

## Results

This study focused on two major cell types of NSCLC, lung adenocarcinoma and squamous cell carcinoma. For each cell type, a comprehensive model was constructed to include the previous AJCC staging system (the 3^rd^ and 6^th^ editions) and the current AJCC 7^th^ edition. The clinical characteristics of the SEER patient population are listed in Table 1, and two external validation cohorts are summarized in Table 2. The bootstrapped model was used to generate a hazard score of each patient in the test data as a blinded validation. The previously determined parameters and cutoffs were used to stratify patients in the original cohort into the three risk groups based on the hazard score of each patient. The prognostic categorization of the comprehensive model was compared with multiple editions of the AJCC staging system. Specifically, the low-risk group defined by the comprehensive model was compared with AJCC stage I; the intermediate-risk group was compared with AJCC stage II and IIIA; whereas the high-risk group was compared with AJCC stage IIIB/IV. Significantly longer survival in the low-risk group or significantly poorer survival in the high-risk group was considered to be an improvement in prognostication using the comprehensive model. The models were constructed by taking the mean of each coefficient from a Cox model fit on 500 bootstrapped samples of each original cohort. This resulted in a total of four models, one for each of the two AJCC staging systems combined with two major NSCLC cell types. These models were tested on the original samples in their entirety, sub-cohorts representative of four major treatment modalities, and two external cohorts.

**Table 1:** Outline of patient clinical characteristics for major histology of non-small cell lung cancer and AJCC staging editions retrieved from SEER database.

	Adenocarcinoma		Squamous	
Variable*	AJCC 3^rd^&6^th^	AJCC 7^th^	AJCC 3^rd^&6^th^	AJCC 7^th^
**Age**				
*Mean Age (σ)*	66.9 (11.4)	67.0 (11.3)	69.0 (9.9)	69.8 (10.2)
**Sex**				
*Male*	75,753 (50.4%)	18,550 (48.3%)	55,794 (66.2%)	12,678 (62.7%)
**Race**				
*API*	10,377 (6.9%)	2,853 (7.4%)	3,877 (4.6%)	885 (4.4%)
*Black*	14,432 (9.6%)	3,620 (9.4%)	10,373 (12.3%)	2,317 (11.5%)
*White*	125,349 (83.5%)	31,953 (83.2%)	70,004 (83.1%)	17,006 (84.2%)
**Tumor Stage**				
*I*	36,052 (24%)	8,295 (21.6%)	21,495 (25.5%)	4,090 (20.2%)
*II*	6,118 (4.1%)	4,661 (12.1%)	4,899 (5.8%)	3,026 (15%)
*IIIA*	11,447 (7.6%)	5,773 (15%)	11,284 (13.4%)	4,497 (22.2%)
*IIIB*	26,905 (17.9%)	3,008 (7.8%)	19,933 (23.7%)	2,435 (12%)
*IV*	69,636 (46.4%)	16,690 (43.4%)	26,643 (31.6%)	6,160 (30.5%)
**Tumor Grade**				
*Grade 1*	11,415 (7.6%)	3,602 (9.4%)	2,559 (3.0%)	464 (2.3%)
*Grade 2*	28,999 (19.3%)	8,637 (22.4%)	22,877 (27.2%)	5,700 (28.2%)
*Grade 3*	45,424 (30.3%)	9,796 (25.5%)	32,380 (38.4%)	7,266 (36%)
*Grade 4*	64,320 (42.8%)	16,391 (42.7%)	26,438 (31.4%)	6,779 (33.5%)

*Sub-stages for stage I and II patients are combined as it was not possible to differentiate between sub-stages for all patients staged with the AJCC 3^rd^ and 6^th^ staging systems. Age is represented as the mean age with the standard deviation in parentheses.

**Table 2:** Outline of patient clinical characteristics for external non-small cell lung cancer validation sets.

	Adenocarcinoma		Squamous
Variable	Director's Challenge Study [21]	MBRCC	MBRCC
**Age** *			
*Mean Age (σ)*	64.4 (10.1)	64.3 (11.3)	67.1 (10.1)
**Sex**			
*Male*	218 (50.3%)	419 (52.8%)	479 (63.2%)
**Race**			
*API*	7 (1.6%)	2 (0.3%)	1 (0.1%)
*Black*	12 (2.7%)	9 (1.1%)	15 (2.0%)
*White*	420 (95.7%)	783 (98.6%)	742 (97.9%)
**Tumor Stage**			
*I*	276 (62.9%)	181 (22.8%)	176 (23.2%)
*II*	95 (21.6%)	48 (6%)	57 (7.5%)
*IIIA*	57 (13%)	74 (9.3%)	111 (14.6%)
*IIIB*	11 (2.5%)	95 (12%)	115 (15.2%)
*IV*	0 (0%)	396 (49.9%)	299 (39.4%)
**Tumor Grade**			
*Grade 1*	60 (13.7%)	62 (7.8%)	22 (2.9%)
*Grade 2*	208 (47.4%)	137 (17.2%)	172 (22.6%)
*Grade 3*	166 (37.8%)	231 (29%)	267 (35.2%)
*Grade 4*	5 (1.1%)	364 (45.8%)	297 (39.2%)

*Age is represented as the mean age with the standard deviation in parentheses.

In the overall studied patient population, earlier stage at diagnosis was significantly related to disease-specific survival in a univariate Cox Proportional Hazards model in both adenocarcinoma and squamous cell carcinoma for each AJCC Staging system (*P*<0.05). In the multivariate analyses AJCC stage, tumor grade, patient age, race, and gender were all significant. Specifically, lower tumor grade, younger age at diagnosis, and being of Asian/Pacific Islander descent were all significantly associated with improved survival (*P*<0.05). Being male or having a later stage at diagnosis was associated with a poorer outcome across all groups. The comprehensive model incorporating all these factors showed significantly improved prognostic categorization when compared with the AJCC staging system, including the latest edition which is detailed below.

The patients were then assigned into one of four treatment categories based on the treatment record in the SEER database. These categories were surgery, radiation, surgery with radiation, and no treatment listed. For simplicity, this determination was based on the presence or absence of any surgical or radiation procedure, regardless of the specific procedure.

### Patient stratification for lung adenocarcinoma (the AJCC 3^rd^ and 6^th^ edition)

**Figure 1:**
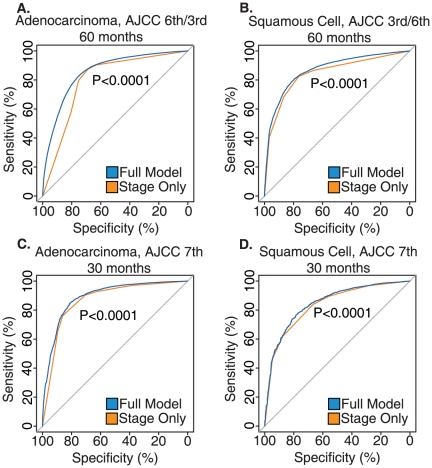
Prediction of survival at 60 months for the AJCC 3^rd^ and 6^th^ Editions (top) and 30 months for the cases converted to the AJCC 7^th^ Edition (bottom) for both lung adenocarcinoma (left) and squamous cell lung cancer (right) using ROC curves. *P*<0.05 indicates that the full model is significantly more accurate in predicting disease-specific survival than tumor stage.

A total of 150,158 lung adenocarcinoma patients staged with the 3^rd^ and 6^th^ AJCC Editions met the criteria for inclusion. Harrell's c statistic was calculated for both the model using stage alone and the comprehensive model using additional covariates. The comprehensive model had a higher C statistic (0.732) compared to the stage only model (0.694), as well as showing better prediction of 5-year survival after the initial treatment in ROC curves (*P*<0.0001, Fig. 1A). A similar improvement was seen for Nagelkerke's R^2^ (0.294 vs. 0.253) and Brier score (0.134 vs. 0.143).

The analysis comparing the performance of each model on treatment subgroups also showed a similar improvement in predictive ability with the comprehensive model. In patients that received surgery without radiation, the comprehensive model had consistently better estimates for Harrell's C (0.768 vs. 0.723), Nagelkerke's R^2^ (0.225 vs. 0.173) and Brier Score (0.206 vs. 0.210). A similar improvement, summarized in Tables 3, 4 and 5, was observed in patients receiving radiation without surgery, surgery with radiation, and those with no treatment listed.

**Table 3:** Harrell's C-statistics from each model for each of the patient cohorts, separated into AJCC coding system, treatment modality, and histology where possible.

	Lung Adenocarcinoma				Squamous Cell Lung Carcinoma			
	*AJCC 3^rd^ &6^th^*		*AJCC 7^th^*		*AJCC 3^rd^ & 6^th^*		*AJCC 7^th^*	
	FM	SO	FM	SO	FM	SO	FM	SO
*All SEER Patients*	0.732	0.694	0.763	0.731	0.722	0.706	0.733	0.717
*Surgery*	0.768	0.723	0.742	0.707	0.762	0.742	0.689	0.670
*Radiation*	0.631	0.608	0.665	0.632	0.647	0.636	0.666	0.658
*Surgery + Radiation*	0.688	0.677	0.696	0.678	0.688	0.674	0.682	0.663
*No Treatment*	0.601	0.542	0.607	0.558	0.582	0.567	0.598	0.580
*MBRCC Cohort*	0.721	0.708	N/A	N/A	0.695	0.681	N/A	N/A
*Director's Challenge Study*	0.687	0.660	N/A	N/A	N/A	N/A	N/A	N/A

FM: full model; SO: AJCC stage only.

**Table 4:** Nagelkerke's R^2^ values from each model for each of the patient cohorts, separated into AJCC coding system, treatment modality, and histology where possible.

	Lung Adenocarcinoma				Squamous Cell Lung Carcinoma			
	*AJCC 3^rd^ & 6^th^*		*AJCC 7^th^*		*AJCC 3^rd^ & 6^th^*		*AJCC 7^th^*	
	FM	SO	FM	SO	FM	SO	FM	SO
*All SEER Patients*	0.294	0.253	0.305	0.274	0.289	0.274	0.246	0.230
*Surgery*	0.225	0.173	0.094	0.073	0.283	0.268	0.064	0.055
*Radiation*	0.107	0.084	0.140	0.115	0.109	0.103	0.120	0.118
*Surgery + Radiation*	0.204	0.178	0.084	0.072	0.201	0.184	0.095	0.089
*No Treatment*	0.066	0.034	0.075	0.044	0.051	0.042	0.065	0.054
*MBRCC Cohort*	0.343	0.311	N/A	N/A	0.244	0.233	N/A	N/A
*Director's Challenge Study*	0.189	0.162	N/A	N/A	N/A	N/A	N/A	N/A

FM: full model; SO: AJCC stage only.

**Table 5:** Brier Scores from each model for each of the patient cohorts, separated into AJCC coding system, treatment modality, and histology where possible.

	Lung Adenocarcinoma				Squamous Cell Lung Carcinoma			
	*AJCC 3^rd^ & 6^th^*		*AJCC 7^th^*		*AJCC 3^rd^ & 6^th^*		*AJCC 7^th^*	
	FM	SO	FM	SO	FM	SO	FM	SO
*All SEER Patients*	0.134	0.143	0.144	0.150	0.119	0.119	0.162	0.161
*Surgery*	0.206	0.210	0.089	0.092	0.186	0.188	0.113	0.114
*Radiation*	0.097	0.099	0.163	0.168	0.102	0.102	0.170	0.172
*Surgery + Radiation*	0.096	0.097	0.153	0.154	0.098	0.099	0.160	0.163
*No Treatment*	0.098	0.101	0.167	0.178	0.078	0.081	0.151	0.152
*MBRCC Cohort*	0.071	0.074	N/A	N/A	0.079	0.081	N/A	N/A
*Director's Challenge Study*	0.163	0.170	N/A	N/A	N/A	N/A	N/A	N/A

FM: full model; SO: AJCC stage only.

**Figure 2:**
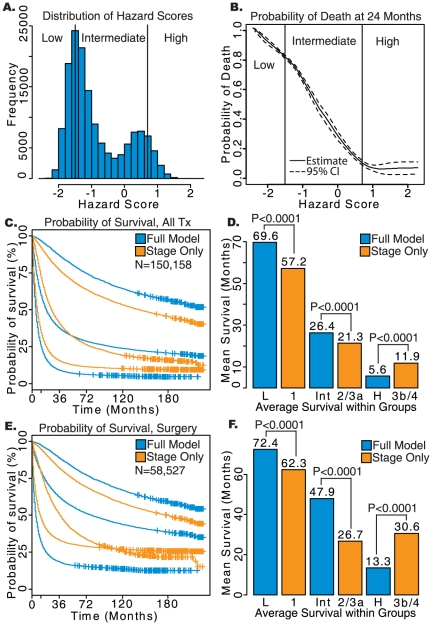
Results of survival analysis on lung adenocarcinoma patients staged using AJCC 3^rd^ or 6^th^ Edition. a) Histogram of Hazard Scores obtained from the comprehensive model. b) Probability of death from lung cancer prior to 24 months based on Hazard Scores calculated using the comprehensive model. c) Kaplan-Meier survival plots for low-, intermediate-, and high-risk groups determined by the comprehensive model (blue) and AJCC staging alone (orange). d) Average survival of each group in months, with log-rank *P*-values shown. L: low-risk; Int: intermediate-risk; H: high-risk defined by the full model. Stage only model contains patient with stage 1, 2, 3a, 3b and 4. e) Kaplan-Meier survival plots for each risk group in patients who received surgery without radiation. f) Average survival for risk groups in patients who received surgery without radiation. L: low-risk; Int: intermediate-risk; H: high-risk. Stage only model contains patient with stage 1, 2, 3a, 3b and 4.

The low-risk group predicted by the comprehensive model survived significantly longer than stage I patients, with an average survival of 69.6 versus 57.2 months (log-rank *P*<0.0001). In addition, the high-risk group predicted by the comprehensive model had a significantly poorer survival than the stage IIIB/IV patient group, with an average survival of 5.6 months compared to 11.9 months (log-rank *P*<0.0001) as shown in Fig. 2C and 2D.

For lung adenocarcinoma patients who received surgery without radiation, the comprehensive model was able to improve upon the prognostic ability of AJCC staging for low-risk patients with an average survival of 72.4 versus 62.3 months (log-rank *P*<0.0001). Patients in the high-risk group had an average survival of 13.3 versus 30.6 months for the comprehensive and stage alone models, respectively (log-rank *P*<0.0001). The intermediate-risk group defined by the comprehensive model showed significantly better prognosis than stage II and III patients (log-rank *P*<0.0001; Fig. 2E and 2F). Similar results were observed for patients receiving other treatment options (results not shown). Specifically, for patients who received both surgery and radiation, radiation without surgery, or no treatment, the comprehensive model could identify patients at higher risk as candidates for adjuvant chemotherapy, whereas it might spare low-risk patients from unnecessarily aggressive treatment.

### Lung adenocarcinoma cases converted to the AJCC 7^th^ edition

**Figure 3:**
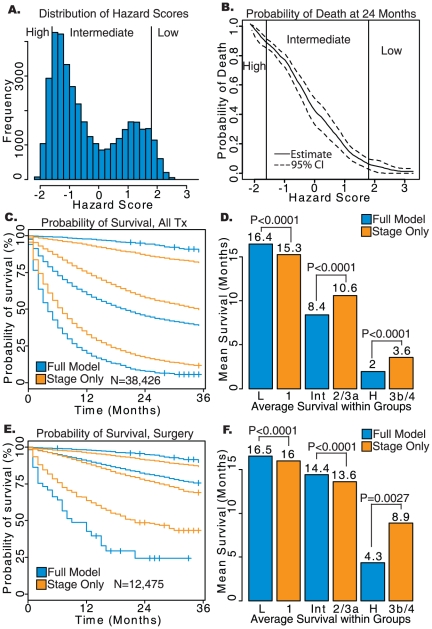
Results of survival analysis on lung adenocarcinoma patients converted to AJCC 7^th^ Edition. a) Histogram of Hazard Scores obtained from the comprehensive model. b) Probability of death from lung cancer prior to 24 months based on Hazard Scores calculated using the comprehensive model. c) Kaplan-Meier survival plots for low-, intermediate-, and high-risk groups determined by the comprehensive model (blue) and AJCC staging alone (orange). d) Average survival of each group in months, with log-rank *P*-values shown. e) Kaplan-Meier survival plots for each risk group in patients who received surgery without radiation. f) Average survival for risk groups in patients who received surgery without radiation. L: low-risk; Int: intermediate-risk; H: high-risk defined by the full model. Stage only model contains patient with stage 1, 2, 3a, 3b and 4.

A total of 38,426 lung adenocarcinoma cases were converted into the AJCC 7^th^ edition. It is important to note that the converted cases represent a much smaller cohort and have shorter follow-up time compared to the AJCC 3^rd^ and 6^th^ Edition cohorts. When considering the entire patient sample, Harrell's C for the comprehensive model versus the stage only model (0.763 vs. 0.731), prediction of survival at 30 months (*P*<0.0001, Fig. 1C), Nagelkerke's R^2^ (0.305 vs. 0.274) and Brier score (0.144 vs. 0.150) were all improved. These effects persisted when considering the four patient sub-cohorts defined by treatment modality, although the performance of both models was similarly decreased when compared to the original staging system. The patient sub-cohort with no treatment listed performed the worst on all three metrics. An improvement in the prognostic categorization similar to that observed in the unconverted cases (the AJCC 3^rd^ and 6^th^ staging) was found for the overall population and specific treatment modalities (Fig. 3). When considering all treatments the low-risk group predicted by the comprehensive model had an average survival of 16.4 months compared to 15.3 months for stage I of the AJCC 7^th^ edition (log-rank *P*<0.0001). Prediction of the high-risk group was also significantly improved with an average survival of 2.0 months for the comprehensive model and 3.6 months for stage IIIB/IV (log-rank *P*<0.0001).

For lung adenocarcinoma patients who received surgery without radiation, the comprehensive model significantly improved prognostication in the low-risk group (16.5 versus 16.0 months, log-rank *P*<0.0001). The high-risk group had an average survival of 4.3 months for the comprehensive model and 8.9 months for stage IIIB/IV (log-rank *P*<0.0001)). The comprehensive model was also able to improve prognostication for both the high and low-risk groups in patients that received both surgery and radiation or no treatment (P<0.05), and in the high-risk group for patients receiving radiation without surgery (*P*<0.0001). Prognostication using the comprehensive model matched or improved non-significantly upon the stage only model in the patient samples which did not achieve significance (results not shown).

### Prognostication of squamous cell lung cancer (the AJCC 3^rd^ and 6^th^ edition)

A total of 84,254 squamous cell lung cancer patients diagnosed with the ACC 3^rd^ and 6^th^ staging system met the inclusion criteria. Performance of both the comprehensive and stage only models were slightly decreased when compared to the adenocarcinoma patients in the overall patient sample. However, there was still an improvement in the overall treatment cohort when using the comprehensive model on Harrell's C (0.722 vs. 0.706), prediction of 5-year survival in ROC curves (*P*<0.0001Fig. 1B), Nagelkerke's R^2^ (0.289 vs. 0.274), but not on Brier score (0.119 vs. 0.119). There was a similar improvement in the sub-cohorts defined by treatment modality, with the comprehensive model performing as well or better than the stage only model in all sub-cohorts. In the overall cohort, the low-risk group defined by the comprehensive model had an average survival of 51.3 months versus 45.7 months in stage I squamous cell lung cancer (log-rank *P*<0.0001). The high-risk group had an average survival of 1.7 months versus 4.7 months in stage IIIB/IV patients (log-rank *P*<0.0001).

**Figure 4:**
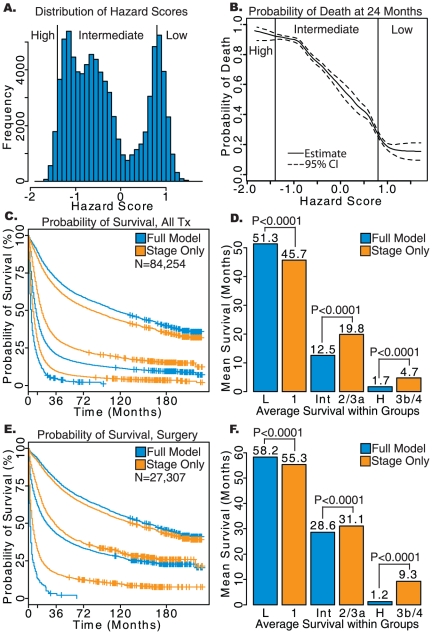
Results of survival analysis on squamous cell lung cancer patients staged using AJCC 3^rd^ or 6^th^ Edition. a) Histogram of Hazard Scores obtained from the comprehensive model. b) Probability of death from lung cancer prior to 24 months based on Hazard Scores calculated using the comprehensive model. c) Kaplan-Meier survival plots for low-, intermediate-, and high-risk groups determined by the comprehensive model (blue) and AJCC staging alone (orange). d) Average survival of each group in months, with log-rank *P*-values shown. e) Kaplan-Meier survival plots for each risk group in patients having received surgery without radiation. f.) Average survival for risk groups in patients who received surgery without radiation. L: low-risk; Int: intermediate-risk; H: high-risk defined by the full model. Stage only model contains patient with stage 1, 2, 3a, 3b and 4.

Similar results were found when comparing only those who received surgical treatment, with the low-risk group predicted by the comprehensive model surviving an average of 58.2 months compared to 55.3 months for stage I patients (log-rank *P*<0.0001), and the high-risk group surviving an average of 1.2 versus 9.3 months in stage IIIB/IV patients (log-rank *P*<0.0001; Fig. 4E and 4F). Similar results were also observed for squamous cell lung cancer patients who received surgery and radiation, radiation without surgery, and no treatment (results not shown) with the comprehensive model improving prognostication among high-risk patients in all three samples (log-rank *P*<0.05), and in low-risk patients for those receiving surgery with radiation or no treatment (log-rank *P*<0.05).

### Squamous cell lung cancer cases converted to the AJCC 7^th^ edition

A total of 20,208 squamous cell lung cancer cases could be converted to the AJCC 7^th^ edition. Prediction was similar or improved when using the comprehensive model on all three metrics and in all treatment cohorts considered, however the difference between the two models was marginal in some cases. The most marked improvement in prediction was in the sub-cohort of patients receiving surgery without radiation. For that group, the comprehensive model outperformed the stage only model on Harrell's C (0.689 vs. 0.670), prediction of survival at 30 months (*P*<0.0001, Fig. 1D), Nagelkerke's R^2^ (0.064 vs. 0.055), and marginally on Brier score (0.113 vs. 0.114).

The low-risk group predicted by the comprehensive model survived an average of 14.7 months, representing a significantly better prognosis than average survival of 13.7 months in stage I patients (log-rank *P*<0.0001). The high-risk group had an average of 1.8 versus 3.0 months when compared to stage IIIB/IV patients (log-rank *P*<0.0001).

**Figure 5:**
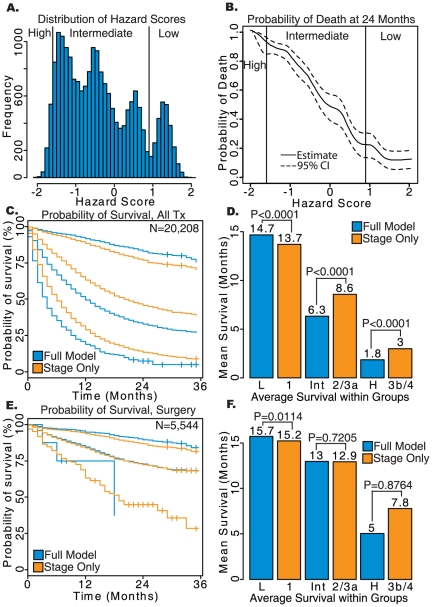
Results of survival analysis on squamous cell lung cancer patients converted to AJCC 7^th^ Edition. a) Histogram of Hazard Scores obtained from the comprehensive model. b) Probability of death from lung cancer prior to 24 months based on Hazard Scores calculated using the comprehensive model. c) Kaplan-Meier survival plots for low-, intermediate-, and high-risk groups determined by the comprehensive model (blue) and AJCC staging alone (orange). d.) Average survival of each group in months, with log-rank *P*-values shown. e) Kaplan-Meier survival plots for each risk group in patients who received surgery without radiation. f.) Average survival for risk groups in patients who received surgery without radiation. L: low-risk; Int: intermediate-risk; H: high-risk defined by the full model. Stage only model contains patient with stage 1, 2, 3a, 3b and 4.

In patients receiving surgery without radiation, the comprehensive model predicted an average survival of 15.7 months for the low-risk group versus 15.2 months for stage I (log-rank *P* = 0.0114). The average survival of the high-risk group did not differ significantly from that of stage IIIB/IV (*P* = 0.8764), due in part to the small sample size and short follow-up, although the comprehensive model showed a non-significant improvement of 5.0 versus 7.8 months. These results are summarized in Fig. 5. In patients treated with radiation without surgery or radiation with surgery, prognostic categorization was improved only in the high-risk group, with an average survival of 2.1 versus 3.2 months and 2.4 versus 6.1 months, respectively, compared to stage alone (log-rank *P* = 0.0136; results not shown).

### Treatment selection for stage I patients

**Figure 6:**
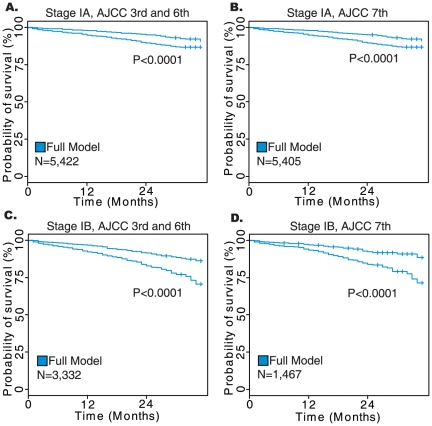
Results of survival analysis on lung adenocarcinoma patients diagnosed with stage IA or IB disease. The Kaplan-Meier plots show the difference between low- and high-risk groups as determined by the comprehensive model. Data on sub-stage was only available for patients staged using the AJCC 6^th^ Edition staging system (2004 and later) and for those patients converted into the 7^th^ Edition.

**Figure 7:**
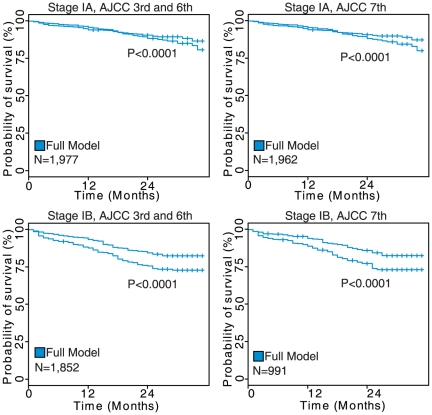
Results of survival analysis on squamous cell lung carcinoma patients diagnosed with Stage IA or IB disease. The Kaplan-Meier plots show the difference between low- and high-risk groups as determined by the comprehensive model. Data on sub-stage was only available for patients staged using the AJCC 6^th^ Edition staging system (2004 and later) and for those patients converted into the 7^th^ Edition.

Patients with stage I cancers who were treated with surgery without radiation were extracted for a further analysis to determine whether the comprehensive model could identify early-stage patients who may benefit from a more aggressive therapy. The stage I cohort was then further separated into stage IA and IB patients, with the coefficients from the comprehensive model being applied in order to test the ability of the additional factors to stratify a relatively homogenous set of patients. High and low-risk group membership was defined relative to the median hazard score for each cohort. For adenocarcinoma the comprehensive model was able to stratify stage IA and IB using both the 3^rd^ and 6^th^ Editions as well as the 7^th^ Edition (log-rank *P*<0.0001) in Kaplan-Meier analyses (Fig. 6). In squamous cell carcinomas the comprehensive model was again able to significantly stratify stage IA and IB patients into high and low-risk groups with both AJCC staging schemes using the model developed on the entire SEER cohort without re-estimation of the parameters (log-rank *P*<0.0001; Kaplan-Meier analyses; Fig. 7). These results demonstrate that the comprehensive prognostic model was able to reliably identify stage I NSCLC patients at higher risk for tumor recurrence. These high risk patients should be considered for adjuvant chemotherapy.

### External Validation

The comprehensive model was also able to improve prognostication in the external validation sets from MBRCC and the Director's Challenge cohort [21]. Patients with both adenocarcinomas (*n* = 794) and squamous cell carcinomas (*n* = 758) with all tumor stages were available from the MBRCC cohort. The Director's Challenge cohort contained only lung adenocarcinoma patients with stage I, II, and III (*n* = 439). The comprehensive model performed consistently better across all three metrics considered when the training models estimated using the SEER cohort was applied to the cohorts from MBRCC and the Director's Challenge study, with the results being consistent across histology in the MBRCC cohort. The comprehensive model appeared to perform much better in the MBRCC cohort. These results are summarized in Tables 3, 4, and 5.

**Figure 8:**
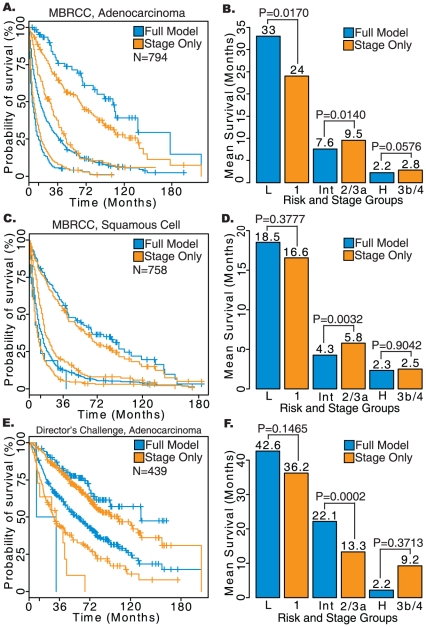
Results of survival analyses performed on patient cohorts from the Director's Challenge Study and the Mary Babb Randolph Cancer Center at West Virginia University.

In the adenocarcinoma cohort from MBRCC, the comprehensive model was able to improve prognostication for the low-risk group (33 versus 24 months, *P* = 0.0170) and borderline significant for the high-risk groups (2.2 versus 2.8 months, *P* = 0.058). The addition of pathological and demographic factors could not significantly improve prognostication in the squamous cell carcinoma patients from the same set (*P*>0.05). In the Director's Challenge cohort which contained only adenocarcinomas, the comprehensive model was able to improve prognostication for the low-risk (42.6 versus 36.2 months) and the high-risk group (2.2 versus 9.2 months), although the results were not significant (*P*>0.05). These results are illustrated in Fig. 8.

## Discussion

Substantial efforts have been made to establish prognostic factors for patients with lung cancer during the last two decades. The traditional prognostic factors are tumor size, vascular invasion, poor differentiation, high tumor-proliferative index, and genetic alterations, including K-*ras* [24], [25] and p53 [26]. With the development of molecular biotechnology, especially high-throughput microarrays, there have been a number of promising studies on lung cancer prognosis by transcriptional profiling [21], [27]–[35]. Although the traditional prognostic factors lack the information about the biological diversity of lung cancer and have not reflected the complexity of molecular mechanisms of these diseases, they are still the most valuable criteria for clinicians to decide the relevant therapies [36]. For instance, Adjuvant! (www.adjuvantonline.com) is a prognostic system for lung cancer, breast cancer, and colon cancer based on traditional pathological features, including age, tumor stage, and grade. It has been independently validated as a reliable aid to clinical decision-making on average breast cancer patients [37]. A study by Birim and others [38] also demonstrated that clinical factors such as respiratory function, comorbidity, and smoking behaviors in addition to tumor stage could be used to refine prognosis in a cohort of NSCLC patients (*n* = 766).

In this study, we sought to investigate the impact of clinical, pathological, and demographic factors on lung cancer survival using a population-based approach. It was found that the addition of pathological and demographic covariates to AJCC staging was able to significantly improve predictive ability in both lung adenocarcinomas and squamous cell carcinomas. These additional variables accounted for previously unexplained variation within and independent of tumor stage, and resulted in a more accurate assessment of the risk for treatment failure when evaluated as integrated prognostic indicators. This effect persisted within multiple treatment modalities.

The comprehensive model was able to improve prediction in stage I surgical adenocarcinoma patients, and was able to produce a significant stratification even within sub-stage IA and IB. Low-risk patients defined by the comprehensive model may not benefit from additional therapies while, conversely, those who are predicted as high-risk may benefit from adjuvant chemotherapy.

The comprehensive model demonstrated significant improvement in clinical prediction over the AJCC 7^th^ staging edition despite smaller sample sizes and shorter follow-up. Furthermore, the external validation results indicate that the comprehensive prognostic model constructed from SEER population data could improve prognosis in multiple local hospitals. These findings show promise for a clinical model for more refined prognosis of NSCLC.

It is important to note that the analysis does not account for the varying quality of treatments between institutions. Median county income was used as a rough surrogate measure for this factor in an unpublished analysis. It was found that higher median county income was significantly associated with improved disease-specific survival, but was omitted from the prognostic model as it is not a prudent metric to guide personalized treatment. Removal of median income as a covariate did not have a significant impact on the overall results or the predictive ability of the model as a whole. An additional limitation of the study was the lack of information on the use of chemotherapy and co-morbidities present at the time of diagnosis [39]. It is expected that inclusion of data found in the linked SEER-Medicare database will more appropriately address these issues and allow for further refinement of the model. In future research, we plan to construct a comprehensive model to estimate treatment benefits of commonly used chemotherapies utilizing the SEER-Medicare data, and to partition patients according to a specific treatment approach. A web-based implementation of this model is currently under development, offering nomograms representing benefits for multiple treatment modalities. We envision that this model could be combined with future clinically validated gene signatures for a more refined assessment of patient risk of treatment failures for a variety of modalities.
