# Correction: Combining Clinical, Pathological, and Demographic Factors Refines Prognosis of Lung Cancer: A Population-Based Study

**DOI:** 10.1371/annotation/506974e6-6764-462c-b7d3-3cbd269c3d6c

**Published:** 2011-03-04

**Authors:** Joseph Putila, Scot C. Remick, Nancy Lan Guo

Figures 2A and 2B were mislabeled; the terms "Low" and "High" should be reversed. 

